# The Printing Trade and the Hospitals

**Published:** 1887-11-12

**Authors:** 


### The Printing Trade and the Hospitals.

We were aware that the men engaged on the printing of The Hospital took a keen interest in its contents and production, and it was, as they know, with regret that the Editors heard that the proprietors had decided, for business reasons, to transfer the printing from Messrs. Whiting and Co. to Messrs. Collingridge. We are informed by the proprietors that they had no knowledge, nor did they realise, that there were differences between society and non-society houses in the printing trade. The decision to transfer the printing was arrived at because of the saving which it would effect in the cost of production. It was done entirely as a matter of business, and in the exercise of the ordinary dealings which transpire between business houses and their customers from time to time. It was with no little astonishment, therefore, that we saw the statement in a contemporary that Messrs. Whiting's workmen, who have hitherto always subscribed to the hospitals through the various funds, had threatened to do otherwise for the future, because the printing of this journal has been removed to a non-society house (Messrs. Collingridge's). It has been stated, but we cannot credit it, that the matter will be taken up at the society's next meeting, and the like resolution be formed in all society houses. We think it right to give publicity to this rumour, believing, as we do, that that section of the working classes who belong to the printing trades will not be likely to refuse to contribute to the Hospital Saturday and Sunday Funds without having a reasonable and justifiable cause for any action they may decide to take as a body, whether they belong to society or non-society houses. No doubt the success of The Hospital has been phenomenal, and this may in one sense account for the disappointment of the men at losing the work, though we should be sorry to think, nor can we indeed believe, that our late printers will on reflection pursue the line of action indicated by our contemporary the *Echo* in its issue of the 3rd inst.

